# The relationship of supportive roles with mental health and satisfaction with life in female household heads in Karaj, Iran: a structural equations model

**DOI:** 10.1186/s12889-021-11656-1

**Published:** 2021-09-08

**Authors:** Nooshin Shadabi, Sara Esmaelzadeh Saeieh, Mostafa Qorbani, Touran Bahrami Babaheidari, Zohreh Mahmoodi

**Affiliations:** 1 Student Research Committee, Alborz University of Medical Sciences, Karaj, Iran; 2 Social Determinants of Health Research Center, Alborz University of Medical Sciences, Karaj, Iran; 3 Non-Communicable Diseases Research Center, Alborz University of Medical Sciences, Karaj, Iran; 4 Endocrinology and Metabolism Research Center, Endocrinology and Metabolism Clinical Sciences Institute, Tehran University of Medical Sciences, Tehran, Iran; 5 Dietary Supplements and Probiotic Research Center, Alborz University of Medical Sciences, Karaj, Iran

**Keywords:** Female household heads, Supportive roles, Satisfaction with life, Mental health, Structural equations

## Abstract

**Background:**

Female household heads are faced with more problems than men due to their multiple concurrent roles. The present study was conducted to determine the relationship of supportive roles with mental health and satisfaction with life in female household heads in Karaj, Iran using a structural equations model.

**Methods:**

The present descriptive-analytical study was conducted on 286 eligible female household heads in Karaj, Iran, in 2020, who were selected by convenience sampling. Data were collected using Vaux’s Social Support, the perceived social support scale, the general health questionnaire (GHQ), and the satisfaction with life questionnaire plus a socio-demographic checklist, and were analyzed in SPSS-16 and Lisrel-8.8.

**Results:**

The participants’ mean age was 43.1 ± 1.7 years. According to the path analysis results, satisfaction with life had the highest direct positive relationship with perceived social support (B = 0.33) and the highest indirect positive relationship with age (B = 0.13) and the highest direct and indirect positive relationship with education and social support (B = 0.13). Also, mental health had a direct negative relationship with satisfaction with life (B = -0.29), an indirect negative relationship with social support, and both a direct and indirect negative relationship with perceived support (B = -0.26).

**Conclusion:**

Based on the results, supportive roles had a negative relationship with mental health; in other words, mental health problems increase as supportive roles decrease. They also had a positive relationship with satisfaction with life in female household heads.

Accordingly, given the status and role of women in the health of family members and the community and their greater vulnerability, further attention and support should be directed toward these women by the government and relevant organizations like establishment of counseling-support centers.

## Background

The present-day social developments have led to changes in the social structure of the family, and one of the most important of these changes is the emergence of single-parent or single-head families [1]. The head of the family signifies the person who has significant authority over the other family members and is responsible for the family’s finances [2]. Female heads of households are women who manage their family affairs due to circumstances such as divorce, immigration, death and desertion or imprisonment of the husband [3].

The number of single-parent families is constantly increasing in the western world, and 60% of women worldwide are currently their family’s breadwinners and 37.5% of households around the world are run by women [4]. This percentage is also increasing in Iran, such that the percentage of female-headed households increased from 9.5% in 2006 to 12.1% in 2011 and 12.7% in 2016. There are currently over 3 million female-headed households in Iran [3].

Khazaeian et al. (2018) from Iran showed that this group of women are faced with problems such as less access to job opportunities, lower levels of education among the women and their children, increased delinquency in their children, and various dimensions of poverty [5]. Being a woman, these household heads are also faced with more socioeconomic problems and have less assets than men [6].

These women are among the most vulnerable social groups and are more exposed to psychosocial stresses due to their multiple and simultaneous roles in managing the household, bringing up and caring for the children, and working outside the home [5, 7, 8]. All these factors result in lower satisfaction with life and mental health in this group compared to their peers. Satisfaction with life suggests mental well-being, which is associated with the health status and death. Many factors affect the sense of well-being and satisfaction with life; e.g., demographic factors, socioeconomic status, physical-mental health, social support, and psychosocial adaptation [9–11].

**Fig. 1:**
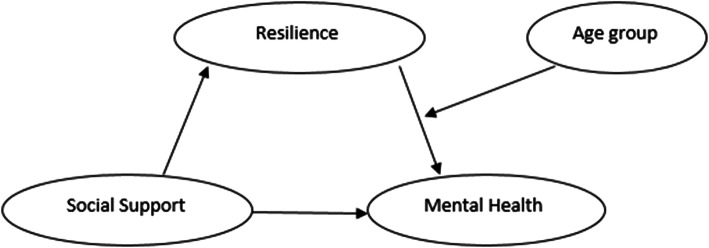
The mediating role of resilience in the association between social support and mental health [13]

Social support is exceptionally important for maintaining good physical and mental health. Overall, it appears that positive social support of high quality can enhance resilience to stress. Mediated by stressors and physical and mental problems alongside boosting people’s knowledge, supportive roles reduce stress, increase survival, and improve quality of life. In other words, this factor provides the most powerful coping force for patients to successfully and easily deal with problems and helps facilitate the endurance of these problems [12]. In one study, Hou et al. (2020) in another population subgroup found that social support is directly and indirectly linked to people’s mental health through flexibility (Fig. 1). Social support via resilience can affect mental health. Resilience is an individual’s capacity to deal with significant adversity and quick recovery. Many studies based on various methodologies and samples have provided robust evidence with respect to the association between social support and resilience [13]. This variable reduces environmental vulnerabilities most likely by affecting the Hypothalamic-Pituitary-Adrenal (HPA) system, the noradrenergic system, and the central oxytocin pathways [12] and subsequently contributes to mental health and satisfaction with life. Although many studies have found a relationship between social support and mental health, the researchers did not find any studies conducted on supportive roles [social and perceived], mental health and satisfaction with life in the population of female household heads. Social support is a multidimensional, latent variable that depends on an individual’s politico-social environment, socialization process and personal values/ethos amongst other factors and perceived social support is perceived adequacy of the available amount of social support [14].

Mental health is not limited to the absence of mental disorders and also includes a state of well-being based on which an individual can actualize their abilities and talents, and adapt to daily life pressures [15].

Given that the health of this vulnerable group is key, the present study was conducted to determine the causal relationship of supportive roles with mental health and satisfaction with life in female household heads by path analysis in Karaj, Iran. We aimed to answer these questions:

1. What is the effect of supportive roles (direct/indirect /total) on satisfaction with life of female heads of households?
2. What is the effect of supportive roles (direct/indirect /total) on mental health of female heads of households?
3. What is the effect of satisfaction with life (direct/indirect /total) on mental health of female heads of households?
4. What is the effect of demographic factors (age, education,) on mental health and satisfaction with life of female heads of households?

Path analysis is considered a causal modeling technique; it can be performed with either cross-sectional or longitudinal data [16].

## Methods

### Study design

The present descriptive analytical study was conducted in 2020 in selected centers of Karaj, the capital of Alborz Province and the fourth most-populated city in Iran and the 22nd most-populated metropolis in the Middle East. On the advice of the Health Deputy of Alborz University of Medical Sciences and based on the information provided by the Integrated Health System of Iran, called the SIB, centers with the largest numbers of referrals of female household heads were selected for this study.

### Study population

Based on the results reported by Chang e al [17]. in their article and considering a correlation of 0.19 between social relations and life satisfaction, first and second types of error of 0.05 and 0.1, respectively, and using the equation below, sample size was determined as 286. Convenience sampling was carried out until the required sample size was achieved.

$$ n={\left(\frac{Z_{\alpha }+{Z}_{\beta }}{C}\right)}^2+3 $$

$$ c=0/5\times \frac{1\mathrm{n}\left(1\kern3.12em +\kern3em r\right)}{1\mathrm{n}\left(1\kern3.12em -\kern3em r\right)} $$

### Inclusion criteria

Women of 20–60 years were included if they were responsible for managing their family for any reason, such as the husband’s death, divorce, imprisonment, or disability, were of Iranian nationality, had minimum reading and writing literacy, gave written consent to participate in the study, and had no physical or mental illness (registered in the system by self-reporting).

### Exclusion criteria

The participants were excluded if they emigrated during the study and were not accessible for completing the questionnaires, returned incomplete questionnaires, or reported using any kinds of psychotropic substances during the study that made them unable to answer the questionnaires.

### Data collection and definition of terms

Data were collected using the following questionnaires: Vaux’s Social Support Scale, the Perceived Social Support Scale, the 28-item General Health Questionnaire (GHQ), the Satisfaction with Life Scale, and a socio-demographic checklist.

Supportive roles were assessed using Vaux’s 23-item Social Support Scale (SS-A) and Zimet’s 12-item Perceived Social Support Scale (PSSS).

By definition, mental health is not limited to the absence of mental disorders and also includes a state of well-being based on which the person can actualize their abilities and talents, and adapt to daily life pressures [15]; in this study, mental health was assessed using the GHQ.

#### Vaux’s social support scale

This questionnaire is theoretically based on Cobb’s (1976) definition of social support [18]. This scale contains 23 items in three domains, including family, friends, and acquaintances. The questionnaire items are scored based on a 4-point Liker scale, from totally agree, to agree, disagree, and totally disagree. Four types of scores are given in this scale, including a score for social support from the family, friends and acquaintances as well as a total social support score, which is the sum of the previous three scores. The validity and reliability of this scale have been determined in previous studies. In the present study, the scale’s reliability was confirmed with a Cronbach’s alpha of 0.83.

#### The perceived social support scale

The multidimensional Perceived Social Support Scale was developed by Zimet et al. and measures three subscales, including support from the family, friends and significant others, by 12 items scored based on a 7-point Likert scale from totally disagree to totally agree. This scale’s total score is calculated as the sum of the scores of all the items. The validity and reliability of this scale were determined in Iran in 2013 by Bagherian et al., and its reliability was confirmed with a Cronbach’s alpha of 0.84 [19]. In the present study, the scale’s reliability was confirmed with a Cronbach’s alpha of 0.82.

#### The general health questionnaire (GHQ)

This 28-item questionnaire was developed by Goldberg & Hiller (1979) with four subscales, including somatic symptoms (items 1–7), anxiety and insomnia (items 8–14), social dysfunction (items 15–21), and severe depression (items 22–28), each with seven items. Scoring is based on a 0 to 3-point scale, with a cut-off point of 23, which means that the higher is the respondent’s score above 23, the worse is their mental health, and the lower is their score, the more ideal their mental health. The validity and reliability of this questionnaire were confirmed in Iran in 2014 by Najarkolaei et al. with a Cronbach’s alpha coefficient of 0.85 [20].

#### The life satisfaction questionnaire (satisfaction with life scale, SWLS)

This scale was developed by Diener et al. (1985) in five domains, each with seven options based on a 1–7-point Likert scale, from totally disagree to totally agree. The sum of the scores is determined in the 5–35 range, and higher scores indicate higher satisfaction [21]. The validity and reliability of this scale were assessed in the Iranian population by Bayani et al. in 2007, and its reliability was confirmed with a Cronbach’s alpha coefficient of 0.83 [22]. In the present study, the reliability of this scale was confirmed with a Cronbach’s alpha of 0.85.

#### Socio-demographic checklist

This checklist contains items about age, education, occupation, marital status, type of marriage, spouse’s status, place of residence, number of children, number of dependents, insurance status, income, and history of illness.

### Procedures

The study began after obtaining the required permissions and a code of ethics from the Ethics Committee of Alborz University of Medical Sciences (IR. ABZUMSREC1397). Due to the COVID-19 pandemic and the need for social distancing, the researcher first visited the selected centers and identified the eligible subjects through the SIB system and then contacted them on their registered phone numbers and explained the study objectives to them and then sent them a consent form to fill out by email or through the centers, if they were willing to take part in the study. Once the consent forms were collected, the questionnaires were emailed to those with internet access (Pars Online), and those without internet access were interviewed on the phone and the researcher filled out their questionnaires for them.

The subjects were ensured about the confidentiality of their data and that it was not obligatory to take part in the study or continue their cooperation, and that they would not be deprived of any health services if they chose not to take part. All methods were carried out in accordance with relevant guidelines and regulations.

### Statistical analysis

**Fig. 2:**
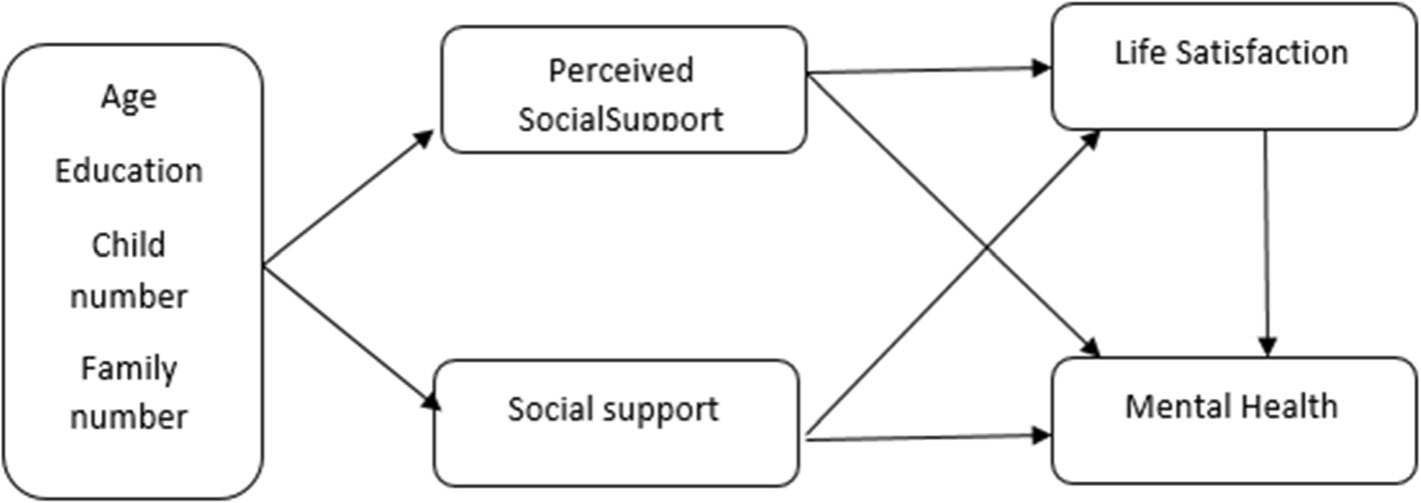
The conceptual model of the relationship between supportive roles, satisfaction with life and mental health

This study examined the fit of a conceptual model for the concurrent relationship of supportive roles (perceived support and social support) with mental health and satisfaction with life (Fig. 2). First, the normal distribution of the quantitative variables was assessed using the Kolmogorov-Smirnov test. Path analysis is an extension of conventional regression that shows not only the direct effects but also the indirect effects of each variable on the dependent variables, and the results can be used to provide a rational interpretation of the relationships and correlations observed. Data were analyzed in SPSS-25 [23] and Lisrel-8.8 [24]. The results were expressed using Pearson’s correlation coefficient for the correlations and in the form of Beta for the path analysis, and the significance level was set at T > 1.96.

## Results

The present study was conducted on 286 eligible female heads of households visiting the selected centers in Karaj, Iran. Participants’ mean age was 43.1 ± 1.7 years and they had 7.05 ± 5.6 years of education on average. The mean score of social support was 62.4 ± 4.9, perceived support was 34.2 ± 10.2, mental health 37.8 ± 10.7, and satisfaction with life 12.1 ± 5.8 (Table 1).

**Table 1:** The socio-demographic characteristics of the participants

Variables [quantitative]		Mean ± SD	Minimum		Maximum
Age [year]		43.1 ± 1.7	20		49
Education		7.05 ± 5.6	0		20
Mental Health	Physical Problems	8.6. ±1.4	social support	family	22.2 ± 2.9
	Anxiety symptoms and sleep disorders	11.6 ± 5.5			
	Social Dysfunction	11.9 ± 4		Friend	18.1 ± 2.5
	Depression	5.7 ± 5.4			
	total	37.8 ± 10.7			
Perceived social support	Family	11.5 ± 4.1			
				acquaintances	22.1 ± 1.6
	Friend	10.8 ± 4.1			
	acquaintances	11.9 ± 3.9			
	total	34.2 ± 10.2		total	62.4 ± 4.9
Variables [qualitative]		F (%)	Variables		F(%)
Number of people in Family	< 2	163 (57)	Occupation	Housewife	85 (29.7)
	≥2	119 (41.6)		Laborer	127 (44.4)
Number of Children	Zero	45 (15.7)		Corporate Job	30 (10.4)
	1	80 (28)			
	2	87 (30.4)			
	3 and above	74 (25.9)			

The results of Pearson’s correlation test showed that education, number of children, social support and perceived support had negative and significant correlations with mental health and positive and significant correlations with satisfaction with life. Among these variables, perceived support and social support had the highest positive and significant correlation with satisfaction with life (*r* = 0.5 and *r* = 0.4, respectively), and perceived support and satisfaction with life had the highest negative correlation with mental health (*r* = − 0.3 and *r* = − 0.39, respectively) (Table 2).

**Table 2:** The correlation between supportive roles, satisfaction with life and mental health in female-headed households

Variable	Age	EDU	NC	NF	LS	Vaux	GHQ	SS
Age	1							
EDU	−0.368^a^	1						
NC	0.406^a^	−0.373^a^	1					
NF	−0.130^a^	0.169^a^	0.428^a^	1				
LS	−0.013	0.373^a^	−0.201^a^	−0.220^a^	1			
Vaux	0.053	0.189^a^	0.016	0.02	0.43^a^	1		
GHQ	0.037	−0.245^a^	0.147^b^	0.096	−0.398^a^	−0.199^a^	1	
SS	0.201^a^	0.174^a^	−0.42	−0.145^b^	0.500^a^	0.519^a^	−.307^a^	1

*Age* Age of female-headed households, *EDU* Education, *NC* Number of Children, *NF* Number of people in Family, *LS* Life Satisfaction, *Vaux* Total Social support, *SS* Total Perceived social support

^a^ Correlation is significant at the 0.01 level (2-tailed)

^b^ Correlation is significant at the 0.05 level (2-tailed)

According to the path analysis results, among the variables related with satisfaction with life, perceived support had the highest positive relationship on the direct path (B = 0.33), age had the highest positive relationship on the indirect path, mediated by perceived support and social support (B = 0.13), and education and social support had a positive relationship with satisfaction with life on both direct and indirect paths (B = 0.38). In other words, higher levels of perceived support, social support, and education were associated with a higher satisfaction with life.

**Fig. 3:**
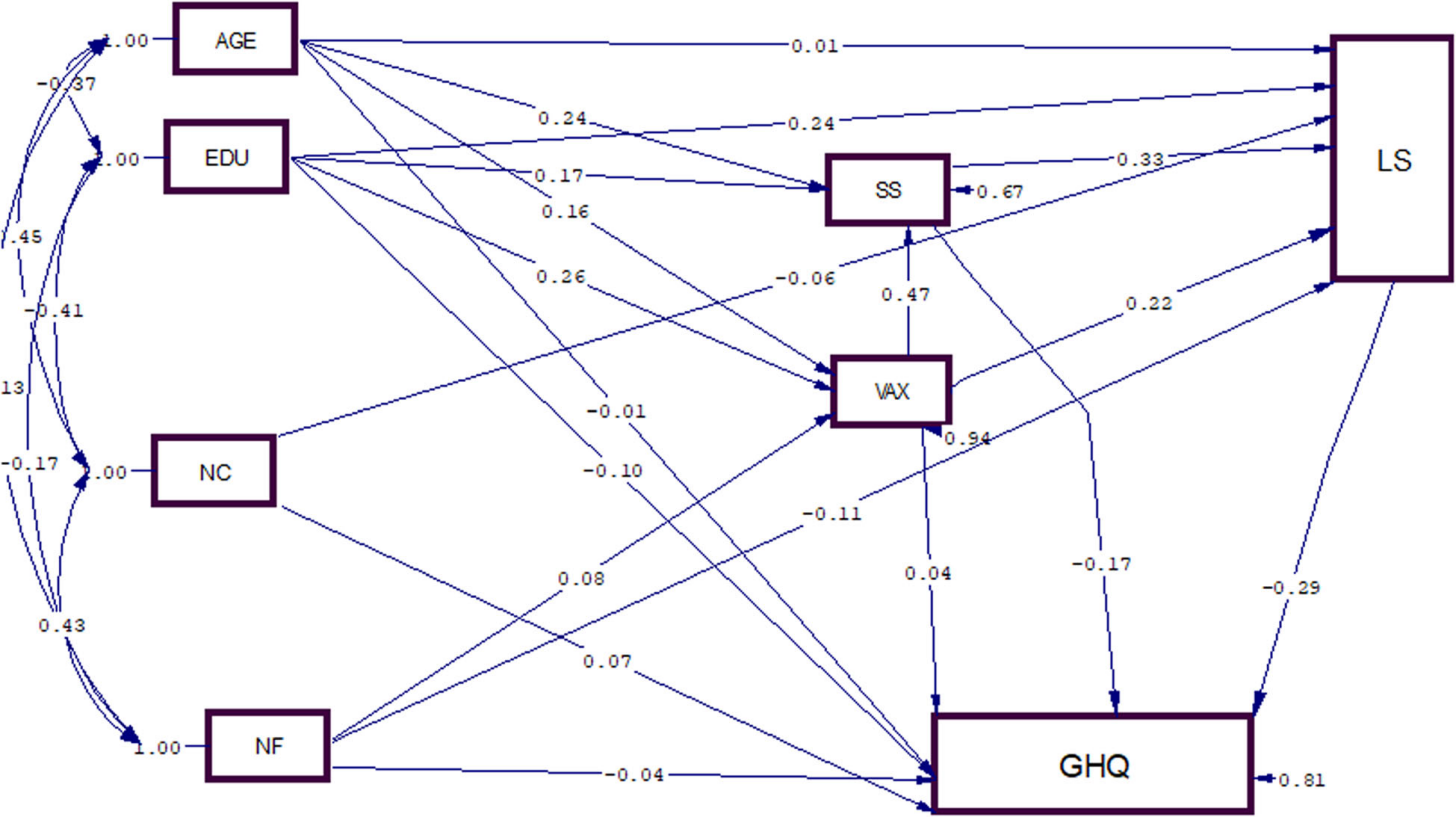
The Full Empirical Model (Empirical Path Model) for the relationships between supportive roles, satisfaction with life and mental health in female-headed households. Age = Age of female-headed households; EDU = Education; NC = Number of Children; NF = Number of people in Family LS = Life Satisfaction Vaux = Total Social support SS = Total Perceived social support

Among the variables related with mental health, satisfaction with life had the highest negative relationship with mental health on the direct path (B = -0.29), social support had the highest negative relationship on the indirect path, mediated through satisfaction with life and perceived support (B = -0.11), and perceived support had the highest negative relationship with mental health on both direct and indirect paths (B = -0.26) (Fig. 3) (Table 3). In other words, lower perceive support, social support, and satisfaction with life were associated with more mental problems.

**Table 3:** The path coefficients for supportive roles with life satisfaction and mental health in female-headed households

Variable		Direct Effect	Indirect Effect	Total Effect
Life satisfaction	Age	–	0.138*	0.138*
	Education	0.24*	0.14*	0.38*
	Perceived Social Support	0.33*	–	0.33*
	Social Support	0.22*	0.16*	0.38*
	Number of Children	−0.06	–	−0.06
	Number of people in Family	−0.11	0.017	−0.09
Mental health	Age	–	−0.09*	−0.09*
	Education	–	−0.073*	−0.073*
	Perceived Social Support	− 0.17*	−0.09*	−0.26*
	Social Support	–	−0.11*	−0.11*
	Number of Children	0.07	0.017	0.087
	Number of people in Family	−0.04	−0.04	−0.08
	Life Satisfaction	−0.29*	–	−0.29*

* means significance

The model fit indices proved favorable and demonstrated the high fit of the model and the rationality of the relationships between the variables based on the conceptual model. Accordingly, the fitted model was not significantly different from the conceptual model (Table 4).

**Table 4:** The goodness of fit indices of the model

Fit Index	X^2^	df	X^2^/df	CFI	GFI	NFI	RMSEA
Model Index	4.59	3	1.53	0.96	0.97	0.92	0.043
Acceptable Range	X2/df < 5			> 0.9	> 0.9	> 0.9	< 0.05

*NFI* Normed Fit Index, *GFI* Goodness of Fit Index, *RMSEA* Root Mean Square Error of Approximation, *X*^*2*^ Chi-Square

## Discussion

Female heads of households experience more stress and psychological problems than men when playing multiple roles at the same time, which can deprive them of satisfaction with life and a favorable mental health [25]. Social support is one of the factors protecting mental health against stresses, and has a significant effect on health, satisfaction with life, and social functioning [26].

Based on the results of the path analysis conducted in this study, among the variables related with satisfaction with life, supportive roles (perceived support and social support) had the highest positive relationship with satisfaction with life. According to the findings, perceived support directly, and social support both directly and indirectly had the highest significant positive relationship with satisfaction with life. In one study, Lu et al. (2018) found that social support influences a range of life experiences and can be linked to satisfaction with life both directly and as a mediator [27]. In another study, Alorani et al. (2018) found that perceived support had a positive and significant relationship with satisfaction with life. They found that by reducing stress and improving social rapport, perceived support also plays a role in increasing satisfaction with life [28]. The structural equations’ path analysis showed that social support can affect the quality of life and the stress-reducing model [29]. There is no doubt that social support has a role in encouraging people to choose healthier lifestyles, and hence, it can affect their quality of life, as well. Then, at times of stress, social support helps the individual not feel alone and makes them sense that they can overcome any problems and reduce their mental pressures. People with sufficient social support at times of stress report better physical and mental health compared to those without adequate support. This better health leads to their greater satisfaction with life and improves their quality of life, too [30, 31].

Based on the path analysis results, among the variables that had a relationship with mental health, satisfaction with life and supportive roles had the highest negative relationship observed. Satisfaction with life was directly, social support indirectly, mediated through satisfaction with life and perceived support, and perceived support both directly and indirectly related negatively with mental health. According to previous studies, satisfaction with life is a predictor of mental health. In other words, higher satisfaction with life is associated with more favorable general and mental health. Receiving more support from other people is also associated with more favorable mental health [32, 33]. The results obtained by Kong et al. (2015) showed that higher satisfaction with life and social support are associated with better mental health. According to their path analysis, social support acts as the mediator in the relationship between appreciation of life and satisfaction with life [34]. People with higher satisfaction with life use more effective and appropriate coping techniques, experience deeper positive emotions, and have better general health [10]. Social support helps the individual deal with tension and stress. In a systematic review study conducted in 2017 on the effect of social support and social capital on the health of female heads of households, social support and its dimensions (emotional, instrumental, and informational) were found to be effective on health, and female heads of households with higher levels of support had a better health status [35].

## Conclusion

Based on the results, supportive roles (social support and perceived support) had a negative relationship with mental health and a positive relationship with satisfaction with life in female heads of households. Accordingly, given the role of women in the health of family members and the society and their greater vulnerability, the government and the relevant organizations are encouraged to address the needs of these women and provide them with more support.

## Data Availability

The data that support the findings of this study are available from the corresponding author upon reasonable request.

## Abbreviations

EDU: Education
NC: Number of Children
NF: Number of people in Family
LS: Life Satisfaction
Vaux: Total Social Support
SS: Total perceived social support
PSSS: Perceived Social Support Scale
SS-A: Social Support Scale

## References

[1] Lebni JY, Gharehghani MAM, Soofizad G, Irandoost SF (2020). Challenges and opportunities confronting female-headed households in Iran: a qualitative study. BMC Womens Health.

[2] Nazoktabar H, Veysi R (2008). Socio-economic and cultural condition of women-headed households in mazandaran province. social welfare. Soc Welfare Quart.

[3] Khodabakhshi-Koolaee A (2020). Comparison of psychological hardiness and resiliency of employed and unemployed female-headed household. J Client Centered Nurs Care.

[4] Rimaz SH, Dastoorpoor M, Vesali Azar Shorbeyani S, Saiepour N, Beigi Z, Nedjat S (2014). The survey of quality of life and its related factors in female-headed households supported by Tehran municipality, Ddistrict 9. IRJE.

[5] Khazaeian S, Kariman N, Ebadi A, Nasiri M (2018). Effect of socio-economic factors on reproductive health in female heads of household: a cross-sectional study in Iran. J Clin Diagn Res.

[6] Espinoza-Delgado J, Klasen S (2018). Gender and multidimensional poverty in Nicaragua: an individual based approach. World Dev.

[7] Gupta A, Mohan U, Tiwari S, Singh S, Singh V, Sc T (2014). Home away from home: quality of life, assessment of facilities and reason for settlement in old age homes of Lucknow, India. Indian J Commun Health.

[8] Bradshaw S, Chant S, Linneker B (2019). Challenges and changes in gendered poverty: the feminization, de-feminization, and re-feminization of poverty in Latin America. Fem Econ.

[9] Thinagaran A, Dass LM. Determinants of life satisfaction among female-headed households in malaysia. Putra Malaysia: University Putra Malaysia. Thesis; 2018.

[10] Humpert S (2013). Gender differences in life satisfaction and social participation. Int J Econ Sci Appl Res.

[11] Veisani Y, Delpisheh A, Sayehmiri K (2015). Health related quality of life in the female-headed households. Int J Epidemiol Res.

[12] Ozbay F, Johnson DC, Dimoulas E, Morgan C, Charney D, Southwick S (2007). Social support and resilience to stress: from neurobiology to clinical practice. Psychiatry.

[13] Hou T, Zhang T, Cai W, Song X, Chen A, Deng G, Ni C (2020). Social support and mental health among health care workers during coronavirus disease 2019 outbreak: a moderated mediation model. PLoS One.

[14] Dambi JM, Corten L, Chiwaridzo M, Jack H, Mlambo T, Jelsma J (2018). A systematic review of the psychometric properties of the cross-cultural translations and adaptations of the multidimensional perceived social support scale (MSPSS). Health Qual Life Outcomes.

[15] Alian Fini F, Ghasemi M (2016). Studying the validity and reliability of the persian version of physical and mental health questionnaire, based on the holistic wellness model. Arak Med Univ J.

[16] Plichta SB, Kelvin EA, Munro BH. Munro’s statistical methods for health care research: Wolters Kluwer Health/Lippincott Williams & Wilkins; 2013. p. 978–1451187946. ISBN-13

[17] Chang PJ, Wray L, Lin Y (2014). Social relationships, leisure activity, and health in older adults. Health Psychol.

[18] Cobb S. Social support as a moderator of life stress. Psychosom Med. 1976;38(5). 10.1097/00006842-197609000-00003. 10.1097/00006842-197609000-00003 981490

[19] Bagherian-Sararoudi R, Hajian A, Ehsan HB, Sarafraz MR, Zimet GD. Psychometric properties of the Persian version of the multidimensional scale of perceived social support in Iran. Int J Prev Med 2013;4(11):1277. PMID: 24404362. PMC3883252 24404362

[20] Najarkolaei FR, Raiisi F, Rahnama P, Fesharaki MG, Zamani O, Jafari MR, et al. Factor structure of the Iranian version of 12-item general health questionnaire. Iran Red Crescent Med J. 2014;16(9). 10.5812/ircmj.11794. 10.5812/ircmj.11794 PMC4270680 25593708

[21] Diener E, Emmons RA, Larsen RJ, Griffin S (1985). The satisfaction with life scale. J Pers Assess.

[22] Bayani AA, Koocheky AM, Goodarzi H (2007). The reliability and validity of the satisfaction with life scale. J Iran Psychol.

[23] Ibm C (2012). IBM SPSS statistics for windows.

[24] Jöreskog KG (1996). LISREL 8 user's reference guide.

[25] Babaiefard A (2014). The social Determinants of female-headed households living pressures in the cities of Kashan & Aran and Bidgol. Soc Welfare Quart.

[26] Fritzell S, Vannoni F, Whitehead M, Burström B, Costa G, Clayton S, Fritzell J (2012). Does non-employment contribute to the health disadvantage among lone mothers in Britain, Italy and Sweden? Synergy effects and the meaning of family policy. Health Place.

[27] Lu M-H, Wang G-H, Lei H, Shi M-L, Zhu R, Jiang F (2018). Social support as mediator and moderator of the relationship between parenting stress and life satisfaction among the Chinese parents of children with ASD. J Autism Dev Disord.

[28] Alorani OI, Alradaydeh MF (2018). Spiritual well-being, perceived social support, and life satisfaction among university students. Int J Adolesc Youth.

[29] Li C, Jiang S, Li N, Zhang Q (2018). Influence of social participation on life satisfaction and depression among Chinese elderly: social support as a mediator. J Commun Psychol.

[30] Nabavi SH, Alipour F, Hejazi A, Rashedi V (2014). Relationship between social support and mental health in older adults. Med J Mashhad Univ Med Sci.

[31] Riahi M, Aliverdinia A, Pourhossein Z (2011). Relationship between social support and mental health. Soc Welfare Quart.

[32] Bakhshipour RA, Peyrovi H, Abedian A (2006). Investigating relationship between satisfaction with life and social support with mental health among freshman students of Tehran university.

[33] Gharibi H, Rostami C, Mohamadian Sharif K, Monqi T (2016). Prediction of defense mechanisms based on the quality of life and perceived social-emotional support in married women. J Health Care.

[34] Kong F, Ding K, Zhao J (2015). The relationships among gratitude, self-esteem, social support and life satisfaction among undergraduate students. J Happiness Stud.

[35] Khazaeian S, Kariman N, Ebadi A, Nasiri M (2017). The impact of social capital and social support on the health of female-headed households: a systematic review. Electr Phys.

